# Mother-Child Interactions and Externalizing Behavior Problems in Preschoolers over Time: Inhibitory Control as a Mediator

**DOI:** 10.1007/s10802-016-0258-1

**Published:** 2017-01-31

**Authors:** Rianne van Dijk, Maja Deković, Tessa L. Bunte, Kim Schoemaker, Mariëlle Zondervan-Zwijnenburg, Kimberly A. Espy, Walter Matthys

**Affiliations:** 1 Department of Child and Adolescent Studies, Utrecht University, P.O. Box 80140, Heidelberglaan 1, 3584 CS Utrecht, The Netherlands; 2 Department of Psychiatry, Brain Center Rudolf Magnus, University Medical Center Utrecht, P.O. Box 85500, Heidelberglaan 100, 3508 GA Utrecht, The Netherlands; 3 Department of Clinical Child & Family Studies, Vrije Universiteit Amsterdam, Van der Boechorststraat 1, 1081 BT Amsterdam, The Netherlands; 4 Department of Methodology & Statistics, Utrecht University, Padualaan 14, 3584 CH Utrecht, The Netherlands; 5 Department of Psychology, University of Arizona, Tucson, AZ USA; 6 Department of Psychology, University of Nebraska-Lincoln, Lincoln, NE USA

**Keywords:** Preschoolers, Affective dyadic flexibility, Maternal negative affect, Inhibitory control, Hyperactivity/impulsivity, Aggressive behavior

## Abstract

Previous research has shown links between parenting and externalizing behavior problems in young children over time. Associations between inhibitory control, one of the executive functions, and externalizing behavior problems are widely established as well. Yet, the role of inhibitory control in the maintenance and change of externalizing behavior problems over time remains unclear. We examined whether inhibitory control could explain the link between mother-child interactions measured on a moment-to-moment timescale and preschoolers’ externalizing behavior problems as reported by teachers. With a sample of 173 predominantly clinically referred preschoolers (76.9% boys) we tested a longitudinal model proposing that affective dyadic flexibility and maternal negative affect predict as well as interact in predicting hyperactive/impulsive behavior and aggressive behavior, with preschoolers’ inhibitory control as a mediator. Our results provide support for this model for preschoolers’ hyperactive/impulsive behavior, but not for aggressive behavior. Hence, inhibitory control is identified as a mechanism linking the content and structure of mother-child interactions to preschoolers’ hyperactivity and impulsivity over time.

**Electronic supplementary material:**

The online version of this article (doi:10.1007/s10802-016-0258-1) contains supplementary material, which is available to authorized users.

Impulsivity, aggressive behavior, and noncompliance are the most frequently reported behavioral problems during early childhood (Keenan and Wakschlag 2004). These types of problems, also referred to as externalizing behavior problems, are the main reason for the clinical referral of preschoolers (Wilens et al. 2002). The presence of externalizing problems at an early age is predictive of maladjustment later in life (Denham et al. 2000). Despite the reported stability of these problems from preschool into the school-aged period (Keenan et al. 2011), recent findings point to changes in externalizing behaviors and related diagnoses (i.e., Attention Deficit Hyperactivity Disorder [ADHD], Oppositional Defiant Disorder [ODD], & Conduct Disorder [CD]) during this period as well (Bunte et al. 2014). By identifying the mechanisms through which externalizing behavior problems develop over time, more specific directions could be provided for intervention programs aimed at reducing these types of problems in preschoolers and preventing the development of more persistent problems over time. In the present study we examined longitudinal links between mother-child interactions, inhibitory control, and preschoolers’ externalizing behavior problems.

## Inhibitory Control and Externalizing Behavior Problems

Executive functions in young children have increasingly gained attention in research on externalizing behavior problems (Schoemaker et al. 2013). Executive functions refer to the cognitive self-regulation of thought, action, and emotion (Séguin and Zelazo 2005). Generally, three different executive functions are identified, namely working memory, shifting, and inhibition (Miyake et al. 2000). In particular, inhibition is an executive function that is considered a requirement for successful self-regulation (Hofmann et al. 2012). Although the terms originally stem from different fields, executive functions and effortful control seem to show many commonalities, and inhibition or inhibitory control is considered an important component of both executive functioning and effortful control (Zhou et al. 2012). In this study we use the term inhibitory control, which refers to processes that enable children to actively inhibit or override a dominant response and initiate a subdominant response. The ability to inhibit a dominant response that is incompatible with a child’s goal is essential for successful self-regulation that develops rapidly during the preschool years (Olson et al. 2009). The capacity to self-regulate is considered a cornerstone for positive development (Shonkoff and Phillips 2000).

Consistent with this view, preschoolers with ADHD or Disruptive Behavior Disorder (DBD) symptoms are found to have weaker inhibitory capacities compared to typically developing preschoolers (Monette et al. 2015; Schoemaker et al. 2013; Schoemaker et al. 2012). Yet, improvements in inhibitory control over time are more distinct in clinically diagnosed preschoolers with ODD/CD or ADHD compared to typically developing children, as they seem to catch up a part of their delay (Schoemaker et al. 2014). It is unclear, however, whether these improvements in inhibitory control are related to a decrease in externalizing behaviors. In their systematic review, Van Lieshout et al. (2013) state that inhibitory control is unrelated to the developmental course of ADHD in children and adolescents, but relatively little studies involved younger children. Therefore, more longitudinal research is needed on the role of inhibitory control in preschoolers’ externalizing behavior problems.

## Inhibitory Control as a Mediator

Rather than merely linking inhibitory control to externalizing behavior problems, it is suggested that children’s inhibitory control may be an important mechanism underlying the often reported link between parenting and preschoolers’ externalizing behavior problems. Examples of parenting dimensions associated with externalizing problems are responsiveness (Johnston et al. 2002), pro-active parenting and parental anger (Denham et al. 2000), psychological and behavioral control (Aunola and Nurmi 2005), and parental hostility (Harold et al. 2013). However, considerably less information is available on *how* parenting is related to preschoolers’ externalizing behavior problems over time (Johnston and Mash 2001). Inhibitory control might be key to better understanding this relation.

Indeed, some longitudinal studies offer support for the role of inhibitory control in explaining the link between parenting and externalizing behavior problems. However, these studies have been conducted in school-aged children (e.g., Valiente et al. 2006) and adolescents (e.g., Eisenberg et al. 2005). Examining the role of inhibitory control in young children seems additionally relevant since executive functions undergo the most rapid development during young childhood (Zelazo and Müller 2002). Unfortunately, longitudinal evidence for the mediating role of young children’s inhibitory control seems inconsistent in different studies in preschool-aged children (Eisenberg et al. 2010; Spinrad et al. 2007). Though, these previous studies are limited by the use of questionnaire measures by the same informant (e.g., mother) to assess both inhibitory control and externalizing behavior problems. Subsequently, Sulik et al. (2015) are among the first to use independent methods for the different constructs measured over time. Based on coded parent-child observations, executive functioning tasks, and questionnaires they report that preschoolers’ executive functioning mediates the relation between early parenting and externalizing behavior problems (i.e., operationalized as conduct problems) in a large community sample.

## The Present Study

The aim of the current study was to further examine the role of inhibitory control in linking parenting and externalizing behavior problems, but in a sample of predominantly clinically referred preschoolers. Second, we extended the work of Sulik et al. (2015) by examining hyperactive/impulsive behavior in addition to aggressive behavior, both of which are considered externalizing behavior problems. Despite reported similarities in inhibitory control in children with different externalizing diagnoses (i.e., ADHD, ODD/CD, or a combination), there appear to be differences as well (Schoemaker et al. 2012). For example, associations between inhibitory control and ODD/CD are more pronounced when motivational demands, such as reward and punishment, are high. This is true for adolescents (Fairchild et al. 2009), school-aged children (Matthys et al. 2004), and even for preschoolers (Schoemaker et al. 2012). Additionally, fewer studies have been conducted on the role of parenting in children’s hyperactive/impulsive behavior as compared to aggression (Johnston et al. 2002; Stormshak et al. 2000). Therefore, we considered hyperactive/impulsive behavior and aggressive behavior as two separate constructs rather than one general construct of externalizing problems.

Third, we used a micro approach in examining dyadic aspects of mother-child interactions. It has been argued that a dyadic interaction is more than just the sum of its parts, and therefore specific dyadic behaviors should be examined (Lunkenheimer and Leerkes 2015). Macro ratings are well-suited for capturing overarching constructs and taking the broader context of behavior into account (Hawes et al. 2013; Heyman et al. 2014) and can even incorporate specific dyadic behaviors (e.g., Kochanska et al. 2008), yielding valuable information to the field. In contrast to macro ratings, however, micro ratings capture the specific sequential relations that characterize interaction patterns (Hawes et al. 2013; Heyman et al. 2014). Micro ratings that capture behaviors as they occur in real time could therefore give a more detailed understanding of dyadic parent-child dynamics (Dishion et al. 2016; Hawes et al. 2013). Consistent with this view, moment-to-moment interaction patterns are thought to reflect the proximal engines of child development (Snyder and Stoolmiller 2002). Hence, children are assumed to develop and maintain externalizing behavior problems through their day-to-day, moment-to-moment interactions with others. Likewise, real-time interchanges are used by clinicians to improve family dynamics (Lunkenheimer et al. 2011). By applying a Dynamic Systems (DS) approach (Granic and Patterson 2006), we were able to identify mother-child interactions based on their affective content, but also by their structural, dyadic pattern. Therefore, a more fine-grained understanding of mother-child interactions and preschoolers’ externalizing behavior problems could be obtained.

## Maternal Negative Affect

Since mothers continue to fulfill the role of primary caregiver in current Western societies (Yeung et al. 2001), it can be assumed that preschoolers often interact with their mothers. In 1983, Maccoby and Martin already pointed out the relevance of studying affective behavior during interactions. Although instances of negative affect during mother-child interactions are common in the preschool years (Keenan and Wakschlag 2000), high levels of maternal negativity towards her child are related to externalizing behavior problems in young children (Cole et al. 2003; Denham et al. 2000; Rubin et al. 2003). Rueger et al. (2011) further propose that parental affect states during interactions may underlie the large variety of parenting dimensions. Effective parent training programs, aimed at reducing externalizing problems in young children, already focus on promoting a positive parent-child relationship through altering parents’ affective responses (e.g., Webster-Stratton 2011). While these previous findings are important, research still requires moment-to-moment assessments to specifically capture parental affect during parent-child interactions to obtain a more detailed understanding of their role in child development (Teti and Cole 2011).

Inhibitory control is suggested to play a key role in explaining the link between maternal displays of affect and preschoolers’ externalizing behavior problems. As argued by Hoffman (2000), for example, maternal negative affect is likely to produce affective overarousal in young children, which poses difficulties for using and developing higher-order cognitive processes such as inhibitory control. In addition to a diminished ability to learn, children might be less motivated to learn from interactions with mothers showing high levels of negative affect (Eisenberg et al. 2005). Concurrent links between the display of maternal negative affect towards children and children’s maladjustment have indeed been explained through poor inhibitory control in preschoolers (Eisenberg et al. 2001), but more longitudinal research is still needed.

## Affective Dyadic Flexibility

In addition to the content of mother-child interactions, interaction patterns can be identified by their dyadic structure. According to the DS theory, a mother and child can be seen as a dyadic system during interactions. The system is self-organizing in the sense that it is characterized by recurrent patterns of behavior to which the mother and child are “attracted” (Granic and Patterson 2006). Therefore, a mother-child dyad tends to stabilize in only a subset of all behavioral patterns it can attain. This refers to the structure of a mother-child interaction (“how”) rather than its content (“what”; e.g., affect).

The structure of an interaction is often specified in terms of flexibility (vs. rigidity). Affective dyadic flexibility refers to the repertoire of affect states available to the dyad, the dyad’s capacity to switch among different states, and the degree to which affect states are evenly distributed across all possible patterns a dyad can attain (Hollenstein et al. 2004). Thus, affectively flexible dyads show a larger range in affect states, switch more among different states, and display more evenly distributed patterns compared to dyads that are low in affective flexibility (i.e., rigid). Those advocating a DS approach argue that the expression of all affect states, including negative ones, are adaptive (Granic et al. 2007). It is the ability of a dyad to flexibly switch among a large range of different patterns that is crucial, as this dyad would also accommodate to contextual demands more easily (Thelen and Smith 1998).

Previous studies on children aged 5 years old and older indeed support this notion. Affective dyadic flexibility during mother-child interactions is linked to fewer adjustment problems and specifically to fewer externalizing behavior problems (e.g., Hollenstein et al. 2004), even in clinically referred children (De Rubeis and Granic 2012; Granic et al. 2007). However, much less is known about the link between affective dyadic flexibility and adjustment in children younger than 5 years of age. In a few studies that have been conducted the findings seem inconclusive. On the one hand, Lunkenheimer et al. (2013) show that lower dyadic flexibility is related to higher levels of problem behavior in 3.5-year-old children. On the other hand, in contrast to the DS theory, two studies report more externalizing problem behavior in preschoolers when mother-child dyads are highly flexible in affect (Lunkenheimer et al. 2011; Van den Akker et al. 2013). These latter findings are in line with suggestions from research with mothers and their infants, who tend to show more negativity during the still-face paradigm when preceded by interactions with high levels of dyadic flexibility (Sravish et al. 2013). The inconsistency between studies may exist because Lunkenheimer et al. (2011) and Van den Akker et al. (2013) used multiple indicators of affective dyadic flexibility and examined externalizing problems specifically, whereas Lunkenheimer et al. (2013) only used one indicator for affective dyadic flexibility in predicting more general behavior problems (i.e., a combined measure of internalizing, externalizing, and child’s negativity).

Hence, the limited evidence available actually seems to indicate that, in contrast to DS theory expectations, higher levels of affective dyadic flexibility during mother-child interactions could be detrimental for preschoolers in terms of the development of externalizing behavior problems. Identifying mechanisms through which affective dyadic flexibility is related to externalizing problem behaviors in preschoolers could help to understand this relation more thoroughly. Children’s inhibitory control might be a mechanism that links higher levels of affective dyadic flexibility to higher levels of externalizing problem behavior. During the preschool years, parents act as external regulators of their children’s affect (Bernier et al. 2010; Calkins et al. 1998), which enables children to gradually develop the ability to self-regulate. Because more affectively flexible mother-child interactions are also less predictable and less stable, this might hamper children from acquiring adequate inhibitory control skills that are needed for the development of children’s self-regulation (Hofmann et al. 2012; Sravish et al. 2013), eventually resulting in more externalizing behavior problems.

## Hypotheses

The aim of our study was to examine whether preschoolers’ inhibitory control mediates the relation between mother-child interactions (both the content and structure) and hyperactive/impulsive behavior and aggressive behavior. Our first two hypotheses were that (1) higher levels of maternal negative affect and (2) higher levels of dyadic flexibility both relate to lower levels of preschoolers’ inhibitory control 9 months later, which in turn predict higher levels of hyperactive/impulsive behavior and aggressive behavior another 9 months later, when controlling for initial externalizing behavior problems.

**Fig. 1:**
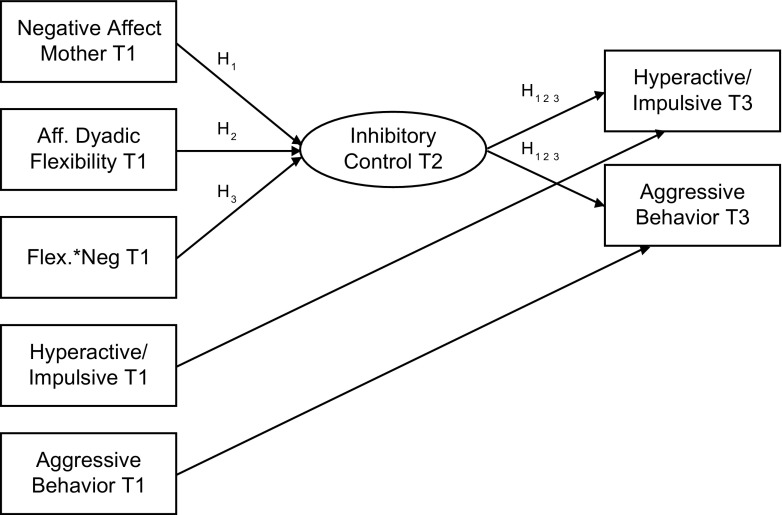
Conceptual representation of proposed model. T1 = first assessment; T2 = 9-month follow-up; T3 = 18-month follow-up; Flex*Neg = interaction of affective dyadic flexibility and negative affect mother. H1, H2, and H3 correspond with our first, second, and third hypotheses in text, respectively

In addition to the proposed main effects for maternal negative affect and affective dyadic flexibility, results by Lunkenheimer et al. (2011) suggest that there is an interplay between the content and the structure of mother-child interactions in explaining externalizing problems as well. Hence, although there are benefits to examining these characteristics of interaction patterns separately, it is also proposed that maternal affect states should be interpreted within the structure in which they are imbedded (Lunkenheimer et al. 2013). Therefore, we explored whether (3) maternal negative affect and affective dyadic flexibility interact in predicting preschoolers’ inhibitory control 9 months later, affecting hyperactive/impulsive behavior and aggressive behavior in preschoolers another 9 months later. A conceptual representation of our proposed model is depicted in Fig. 1.

## Method

### Participants

In the current study we used a sample of 173 mother-child dyads, including clinically referred children (78%) and typically developing children (22%). The sample is part of a larger, longitudinal project (Bunte et al. 2014; Schoemaker et al. 2012; Schoemaker et al. 2014), including three assessments with a 9-month-interval. Children were referred by general practitioners, well-baby clinics, and pediatricians for clinical and psychological assessment to the Outpatient Clinic for Preschool Children with Behavioral Problems, Department of Child and Adolescent Psychiatry, University Medical Center Utrecht (UMCU). For inclusion in the study, referred children had to score at or above the 90th percentile of the Attention problems scale or Aggression scale of either the Children’s Behavioral Checklist (CBCL/1.5–5) or Caregiver-Teacher Report Form (C-TRF/1.5–5; Achenbach and Rescorla 2000). Typically developing children, who were recruited at elementary schools and daycare centers, were excluded when they scored at or above the 90th percentile of either of these scales.

From the original sample (*N* = 251), the following children were excluded to form the current sample: Children of whom observational data was not available due to missing or damaged materials (11.9%); children who were observed in interaction with their father (6.0%) or grandmother (0.4%) instead of their mother; children diagnosed with a disorder other than ADHD, ODD, or CD either at the first or third assessment (2.0%); children with an IQ below 80 (1.2%), as assessed by the average score of the Raven Color Progressive Matrices (Raven et al. 1998) and Peabody Picture Vocabulary Test-III-NL (Dunn and Dunn 2005; Schlichting 2005); children who dropped out of the study after the first or second assessment (6.0%); children with missing C-TRF/1.5–5 scores at the 18-month follow-up (3.2%); and children who had not participated in at least 2 out of 3 inhibitory control task at the 9-month follow-up (0.8%). There were no significant differences between the sample used in this study and children who either dropped out of the study or those who were excluded because of missing C-TRF/1.5–5 scores at T3 or inhibitory control scores at T2, in terms of age, sex, IQ, hyperactive/impulsive behavior as reported by teachers, and inhibitory control scores all measured at T1.

In the current study sample (*N* = 173; 76.9% boys), children’s ages ranged from 42 to 66 months (*M* = 54.76, *SD* = 7.63) at T1, from 50 to 76 months (*M* = 63.72, *SD* = 7.68) at T2, and from 59 to 86 months (*M* = 72.87, *SD* = 7.62) at T3. One-hundred nine of the children were diagnosed with ADHD (*n* = 44), ODD/CD (*n* = 27), or both (*n* = 38). Children were diagnosed on the basis of the strict application of the DSM-IV-TR criteria (American Psychiatric Association 2000), as further described in Schoemaker et al. (2014). Child psychiatrists and clinical child psychologists reached consensus using the following diagnostics: (1) scores on the Attention Problems scale and the Aggression scale of the CBCL/1.5–5 and the C-TRF/1.5–5 (Achenbach and Rescorla 2000); (2) symptoms reported on the Kiddie Disruptive Behavior Schedule (Keenan et al. 2007); (3) scores on the Child Global Assessment Schedule (C-GAS; Shaffer et al. 1983); and (4) the child’s behavior as observed with the Disruptive Behavior Diagnostic Observation Schedule (Bunte et al. 2013a; Wakschlag et al. 2008a; Wakschlag et al. 2008b).

Another 26 referred children, not initially diagnosed, but scoring above the 90th percentile on either the Attention problems scale or Aggression scale (Achenbach and Rescorla 2000), as well as 38 typically developing children were also part of the study in order to increase the variability in outcome measures. Children had an average IQ of *M* = 104.42, with *SD* = 11.36. With regard to the mothers’ education levels, 1.7% had ‘no completed education’, 1.7% completed primary school, 33.0% completed high school, 28.9% completed vocational school and 34% completed (applied) university. The fathers’ education levels followed a similar distribution.

Prior to the study none of the children received medication for their behavioral problems. After the first assessment, 58 children (33.5%) received psychopharmacotherapy, of which most were prescribed methylphenidate (*n* = 54), one risperidone (*n* = 1), and others switched from methylphenidate to atomoxetine after the second assessment (*n* = 3). If children received methylphenidate parents were asked to withhold their child’s medication for 48 h prior to the follow-up assessment. Also, 97 families (56.1%) received a form of psychosocial treatment: Individual parent counseling at home (*n* = 26) or at the outpatient clinic (*n* = 72) and/or participation in the Incredible Years Parent Program (*n* = 7; Webster-Stratton 2011).

### Procedure

Each child’s intellectual functioning and executive functions were assessed over the course of a single morning; a fixed order of tasks was maintained and lasted about 2 h, including breaks (Schoemaker et al. 2012). Executive functioning tasks were administered on a computer. The assessment also included a mother-child observation (i.e., DB-DOS; Bunte et al. 2013a; Wakschlag et al. 2008a, 2008b), and a parent interview (i.e., K-DBDS; Bunte et al. 2013b; Keenan et al. 2007). Parents and teachers were asked to fill in questionnaires. The intellectual assessment was only administered during the first session. The DB-DOS took place at both the first and third assessment. All other measures were administered three times with an interval of 9 months. Written informed consent was obtained from parents before participating in the study. The study protocol was approved by the Medical Ethical Review Committee of the UMCU. Parents received a nominal financial compensation for their participation and children received two small gifts.

### Measures

#### Affective Dyadic Flexibility

Observations of the mother-child interactions recorded at the first assessment were used to measure affective dyadic flexibility. Interactions were initially taped in order to administer the DB-DOS (Wakschlag et al. 2008a, 2008b). The DB-DOS is a 50-min structured laboratory observation, divided into three interactional settings: One parent context followed by two examiner contexts. Our focus was on the first part of the observation in which the mother and child interacted during tasks that were designed for active parent engagement. During the interaction, attractive toys were available at the table behind the mother and child. Mothers were instructed that their children were not allowed to touch or play with the toys, creating a possible stressor. Mothers also had to instruct children what task to do and when to switch tasks (i.e., based on a bell rang by the examiner behind a one-way mirror). In total, 7 min were coded on a moment-to-moment timescale (i.e., every 5 s), including 3 min of coloring, 2 min of clean-up, and 2 min of puzzling. This way we could capture the characteristics of mother-child interaction over a range of different situations.

Based on facial expressions and voice tone, interactions were coded using the following affect codes of the Relationship Affect Coding System (RACS; Peterson et al. 2009): (1) Anger/Disgust: Open anger, irritation/constrained anger or the expressions of being repulsed and disgusted by something someone has said or done. (2) Distress: Decrease in energy and a passive, resigned countenance. It may also resemble fear, sound like whining or appear as sadness (e.g., crying). (3) Ignore: Children turning away from their mother and disregarding her directions. Mothers paying no attention to their children’s pleas for attention, rewards, or social interaction. (4) Validation: Actively communicating that he/she is listening, tracking and is engaged in what the other person is saying or doing. Also, compliments in combination with a physical orientation towards the other person and a display of positive affect. (5) Positive affect: The display of happiness and surprise attributes (e.g., caring, laughter, enjoyment), characterized by a general appearance of positive emotion. (6) Neutral: Non-emotional in both content and voice tone.

Both the affect state of the mother and that of the child were coded by the first author and a trained graduate student. Both coders were unaware of children’s symptomology or diagnosis. They showed a good inter-rater reliability, with an agreement rate of 85.0% and a κ_weighted_ of 0.62 (Sim and Wright 2005), covering 13.9% of the total amount of coded data.

All possible affect states a system can attain were represented by a 6-by-6 state space grid (SSG; Hollenstein 2007), using the software program GridWare 1.15a (Lamey et al. 2004). A SSG allows for the visualization and modelling of dyadic interaction patterns as they unfold on a moment-to-moment timescale. The child’s affect states are plotted along the *y*-axis and the mother’s along the *x*-axis. As a result, the trajectory made up of sequential dyadic states (i.e., the combination of mother’s and the child’s affect states represents a unique dyadic state) can be mapped onto the grid.

**Fig. 2:**
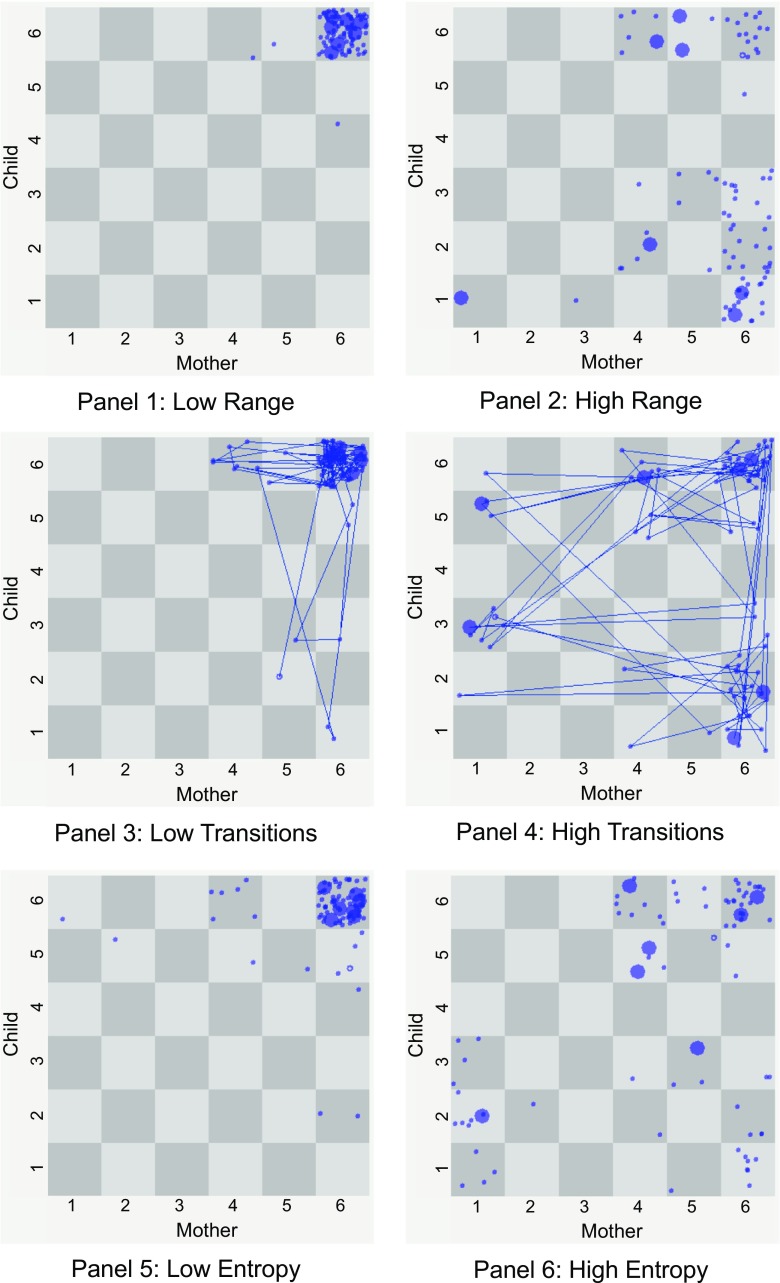
SSG’s on the left are examples of dyads with low flexibility measures, and those on the right show trajectories with high flexibility measures. Each number represents a different affect state

Based on previous studies, affective dyadic flexibility encompassed three measures: (1) the range of affect states visited by dyads (range), (2) the average number of transitions between states per minute (transitions), and (3) the average of all individual cell mean durations (duration entropy; Hollenstein 2007). A high level of flexibility is characterized by a large range of affect states, a high number of transitions, and high levels of duration entropy (i.e., a more even distribution of time spent in different affect states). Two examples (i.e., low versus high flexibility dyad) of each measure are depicted in Fig. 2.

#### Maternal Negative Affect

The observations were used to measure the total amount of maternal negative affect, in which Anger/Disgust, Distress, and Ignore were identified as negative. The number of events in which mother displayed any type of negative affect was summed, divided by the total number of coded events, and then multiplied by 100. This resulted in a percentage of negative affect displayed by the mother in each mother-child dyad.^1^

#### Inhibitory Control

Children’s inhibitory control was measured at the second assessment through three executive function tasks: Shape School Inhibit, Modified Snack Delay, and Go-No-Go (Schoemaker et al. 2012). In the computerized Shape School-Inhibit task, children were asked to name the color of cartoon figures with happy faces, but suppress this color naming when a cartoon with a frustrated or sad face appeared. The number of correct answers was divided by the total number of 18 trials.

The Modified Snack Delay, is a relatively newly developed task that incorporates the motivational aspect from the original Snack Delay paradigm (Kochanska et al. 1996) with the motor-inhibitory control demands of the NEPSY Statue task (Korkman et al. 1998). While being videotaped, children were told to stand still like a snowman while placing both hands on a mat, without talking or moving. A bell and a glass with a treat underneath was placed in front of the child. The examiner told the child that they could move again and eat the treat when the examiner rang the bell. The task lasted for 4 min, during which the child was progressively distracted by various activities by the examiner, such as dropping a pencil, knocking under the table, culminating in the examiner leaving the room for 90 s. Trained raters rated hand movements of the children every five seconds and with three categories (0 = *no movement*, ½ = *some movement*, 1 = *lots of movement*) for every event*.*

In the computerized Go-No-Go task children had to press a button when a fish appeared on their screen (i.e., Go-stimuli, 75%), but they needed to suppress the urge to press whenever a shark appeared (i.e., No-Go stimuli, 25%). Incorrect No-Go trials were subtracted from the number of correct Go-trials, thus, a higher score indicates a better performance on the task.

Previous research reports an adequate test-retest reliability (0.71) for the Shape School-Inhibit task. The Modified Snack Delay and the Go-No-Go both showed a good test-retest reliability (>0.80; Schoemaker et al. 2012). For the purpose of the current study, inhibitory control measured at the second assessment was represented by a latent variable, based on the three executive functioning tasks described above.

#### Externalizing Behavior Problems

Preschoolers’ externalizing behavior problems were measured at the first and third assessment using the C-TRF/1.5–5 Attention problems (which we refer to as hyperactive/impulsive behavior since most items refer to hyperactivity and impulsivity) scale (9 items, Chronbach’s α = 0.90) and Aggression scale (25 items, Chronbach’s α =0.96). Kindergarten and daycare teachers reported on children’s externalizing problems using a 3-point scale (0 = *true*, 1 = *somewhat/sometimes true*, 3 = *very/often true*; Achenbach and Rescorla 2000). T-scores on the Attention problems scale and Aggression scale represented the dependent variables.

### Data Analytic Plan

The hypothesized model was tested using a path analysis in *Mplus* 7.4 (Muthén and Muthén 2015). A maximum likelihood estimator with robust standard errors (MLR) was used to account for the non-normally distributed data. Testing the hypothesized model included several steps. First, the measurement models for both inhibitory control and affective dyadic flexibility were tested. The factor scores of affective dyadic flexibility were saved in order to compute an interaction term with maternal negative affect for subsequent analyses. Centered scores were used to compute the interaction term.

Second, the model fit of the hypothesized mediation model was examined. This model proposed that maternal negative affect, affective dyadic flexibility, and their interaction at T1 would predict child inhibitory control at T2, which would in turn affect child hyperactive/impulsive behavior and aggressive behavior at T3. In this model, we also identified possible direct effects from the predictors at T1 and dependent variables at T3, in order to circumvent possible bias of estimation of conditional indirect effects (Hayes and Preacher 2013). We controlled for initial hyperactive/impulsive behavior and aggressive behavior at T1. Also, received medication (yes/no) and psychosocial treatment (yes/no) after the first or second assessment were entered as control variables. Because inclusion of these latter control variables did not alter the patterns of our findings, they were omitted from the analyses.

We evaluated the model fit with the Comparative Fit Index (CFI), the Tucker-Lewis Index (TLI) and the Root Mean Square Error of Approximation (RMSEA). According to Byrne (2012) CFI and TLI > 0.90 represent an acceptable model fit, with >0.95 indicating a good fit for both indices. Obtaining a RMSEA value <0.08 is indicative of an acceptable fit, but <0.05 is indicative for a good model fit. To determine significance of the estimates, an α-level of 0.05 was used.

Third, we ran our model again using Bayesian estimation for a robustness check of the indirect effects. As computing indirect effects involves multiplying (assumed) normally distributed variables of which the product in itself is not normally distributed, this can yield inaccurate confidence limits and significance tests (MacKinnon et al. 2004). Bayesian estimation has advantages in validating indirect effects in studies with relatively small sample sizes in comparison to other methods (Yuan and MacKinnon 2009). Since the results from the analysis using a Bayesian estimator yielded similar results regarding the direction of the indirect effects, specifications of our Bayesian analysis and results are shown in the Supplementary material.

## Results

### Descriptive Statistics

Descriptive statistics (means, standard deviations and intercorrelations) for each of the study variables are depicted in Table 1. Associations were in the expected direction. Small negative correlations emerged between maternal negative affect at T1 and inhibitory control measures at T2. All measures of affective dyadic flexibility at T1 were also negatively related to all inhibitory control measures at T2, with correlations varying from small to moderate. In turn, inhibitory control measures at T2 were negatively associated with both hyperactive/impulsive behavior and aggressive behavior at T3. There were also small and positive relations between flexibility measures and externalizing problems at T1 and T3, indicating that higher levels of maternal negative affect and higher levels of affective dyadic flexibility at T1 relate to higher levels of both types of externalizing problems measured at T1 and T3. Correlations between T1 and T3 externalizing behavior problems revealed stability levels of moderate and strong effect sizes for hyperactive/impulsive behavior and aggressive behavior, respectively. Also noteworthy were the strong, positive associations between maternal negative affect and affective dyadic flexibility measured at T1.

**Table 1:** Descriptive statistics and pearson correlations of the study variables (*N* = 173)

	1	2	3	4	5	6	7	8	9	10	11
1. Range T1	-										
2. Entropy T1	0.81^**^	-									
3. Transitions T1	0.70^**^	0.82^**^	-								
4. Negative affect mother T1	0.61^**^	0.55^**^	0.46^**^	-							
5. Shape School Inhibit T2	-0.22^**^	-0.27^**^	-0.28^*^	0.22^**^	-						
6. Snack Delay T2	-0.41^**^	-0.39^**^	-0.35^**^	0.27^**^	0.31^**^	-					
7. Go-No-Go T2	-0.25^**^	-0.32^**^	0.33^**^	-0.33^**^	0.39^**^	0.42^**^	-				
8. Hyperactive/impulsive T1	0.31^**^	0.23^**^	0.21^**^	0.25^**^	-0.25^**^	-0.28^**^	-0.21^**^	-			
9. Aggressive behavior T1	0.28^**^	0.23^**^	0.19^*^	0.28^**^	-0.14^*^	-0.22^**^	-0.19^*^	0.66^**^	-		
10. Hyperactive/impulsive T3	0.33^**^	0.29^**^	0.21^**^	0.31^**^	-0.27^**^	-0.41^**^	-0.24^**^	0.41^**^	0.41^**^	-	
11. Aggressive behavior T3	0.28^**^	0.28^**^	0.22^**^	0.39^**^	-0.14	-0.22^**^	-0.22^**^	0.34^**^	0.56^**^	0.63^**^	-
*M*	8.99	1.12	5.02	4.41	5.29	2.93	2.82	62.79	60.80	59.61	57.79
*SD*	2.55	0.41	1.37	5.70	1.36	1.13	0.82	11.52	10.96	9.29	8.63
*Min.*	4.00	0.13	0.86	0.00	0.00	0.00	0.14	50.00	50.00	50.00	49.00
*Max.*	17.00	2.24	8.55	30.95	6.00	4.75	3.70	100.00	94.00	100.00	92.00

**p < 0.01 *p < 0.05

### Model Test

#### Measurement Model

Standardized factors loadings of the constructs affective dyadic flexibility and inhibitory control were examined in order to validate the hypothesized measurement model. Affective dyadic flexibility showed adequate factor loadings of 0.83, 0.85, and 0.97 for range, transition, and duration entropy, respectively. With a factor score determinacy of 0.98, our estimated factor scores were validated and could be saved for further analyses (Schreiber et al. 2006). Saved scores were used to compute the interaction term (affective dyadic flexibility*maternal negative affect). Regarding inhibitory control adequate factor loadings of 0.53, 0.56, and 0.73 were obtained for the *Shape School Inhibit,* the *Modified Snack Delay*, and the *Go-No-Go*, respectively.

#### Structural Equation Model

Based on the *Mplus* modification indices, and because both the Shape School Inhibit and the Go-No-Go were computerized tasks, measurement errors of these constructs were allowed to correlate in the final model. Another justification for this correlation can be found in that both tasks require cool cognitive skills in contrast to more hot cognitive skills (Hongwanishkul et al. 2005), which are needed in the Modified Snack Delay task. The hypothesized model was found to adequately fit the data, as *χ* ^*2*^ (17) = 22.63, *p* = 0.162, RMSEA =0.04, 95% CIs [0.00, 0.09], CFI = 0.98, TLI = 0.95. Parameter estimates, their standard errors, and associated betas are depicted in Table 2.

**Table 2:** Model estimates of coefficients (Maximum likelihood robust estimation; *N* = 173)

		*b*	*SE* of *b*	β	*p*
Paths a (predictor ➔ mediator)					
Aff. dyadic flexibility T1	➔ Inhibitory control T2	**-0.12**	**0.05**	**-0.40**	**0.020**
Negative affect mother T1	➔ Inhibitory control T2	**-0.05**	**0.02**	**-0.48**	**0.017**
Flex*Neg T1	➔ Inhibitory control T2	**0.02**	**0.01**	**0.38**	**0.016**
Paths b (mediator ➔ outcome)					
Inhibitory control T2	➔ Hyperactive/impulsive T3	**-7.10**	**2.65**	**-0.46**	**0.007**
Inhibitory control T2	➔ Aggressive behavior T3	-1.78	1.84	-0.13	0.334
Paths c’ (predictor ➔ outcome)					
Aff. dyadic flexibility T1	➔ Hyperactive/impulsive T3	-0.23	0.44	-0.05	0.598
Negative affect mother T1	➔ Hyperactive/impulsive T3	-0.09	0.25	-0.06	0.711
Flex*Neg T1	➔ Hyperactive/impulsive T3	0.11	0.07	0.17	0.106
Aff. dyadic flexibility T1	➔ Aggressive behavior T3	0.03	0.35	0.01	0.934
Negative affect mother T1	➔ Aggressive behavior T3	0.22	0.20	0.15	0.263
Flex*Neg T1	➔ Aggressive behavior T3	0.04	0.06	0.07	0.502
Stability measures					
Hyperactive/impulsive T1	➔ Hyperactive/impulsive T3	**0.24**	**0.06**	**0.30**	**0.000**
Aggressive behavior T1	➔ Aggressive behavior T3	**0.35**	**0.06**	**0.46**	**0.000**
Covariances					
Aff. dyadic flexibility T1	↔ Hyperactive/impulsive T1	**5.87**	**1.93**	**0.25**	**0.002**
Negative affect mother T1	↔ Hyperactive/impulsive T1	**16.97**	**6.01**	**0.26**	**0.005**
Aff. dyadic flexibility T1	↔ Negative affect mother T1	**6.63**	**1.14**	**0.57**	**0.000**
Aff. dyadic flexibility T1	↔ Aggressive behavior T1	**5.47**	**1.72**	**0.24**	**0.001**
Negative affect mother T1	↔ Aggressive behavior T1	**19.52**	**5.24**	**0.31**	**0.000**
Hyperactive/impulsive T1	↔ Aggressive behavior T1	**83.91**	**12.39**	**0.66**	**0.000**
Hyperactive/impulsive T3	↔ Aggressive behavior T3	**26.92**	**6.36**	**0.53**	**0.000**

Significant estimates are in boldface. *T1* first assessment; *T2* 9-month follow-up; *T3* 18-month follow-up; Flex*Neg = interaction term between maternal negative affect and affective dyadic flexibility. Paths a. b. and c’ refer to the commonly used denotations for the different paths between the predictor, mediator and outcome in mediation models. *R* ^*2*^ = 0.33 for hyperactive/impulsive behavior. *R* ^*2*^ = 0.36 for aggressive behavior

First, the results supported the hypothesis that higher levels of maternal negative affect (H1) relate to lower levels of preschoolers’ inhibitory control 9 months later, which in turn predict higher levels of hyperactive/impulsive behavior another 9 months later. The indirect effect of maternal negative affect on hyperactive/impulsive behavior was statistically significant, as *B* = 0.36, *SE B* = 0.17, β = 0.22, *p* = 0.036. However, no support was found for such an indirect effect on aggressive behavior, as inhibitory control was not related to elevated levels of aggressive behavior, as *B* = 0.09, *SE B* = 0.09, β = 0.06, *p* = 0.359.

Second, similar results appeared for affective dyadic flexibility (H2): Higher levels of affective dyadic flexibility were associated with lower levels of inhibitory control 9 months later, which was predictive of more hyperactive/impulsive behavior another 9 months later. This indirect effect was statistically significant, as *B* = 0.82, *SE B* = 0.41, β = 0.18, *p* = 0.046. Again, this was not the case for aggressive behavior, as *B* = 0.21, *SE B* = 0.20, β = 0.05, *p* = 0.315. Whereas significant correlations existed between maternal negative affect and affective dyadic flexibility at the first assessment, and hyperactive/impulsive behavior and aggressive behavior at the third assessment (see Table 1), these direct relations were non-significant in the structural equation model that included inhibitory control and controlled for initial behavior problems.

**Fig. 3:**
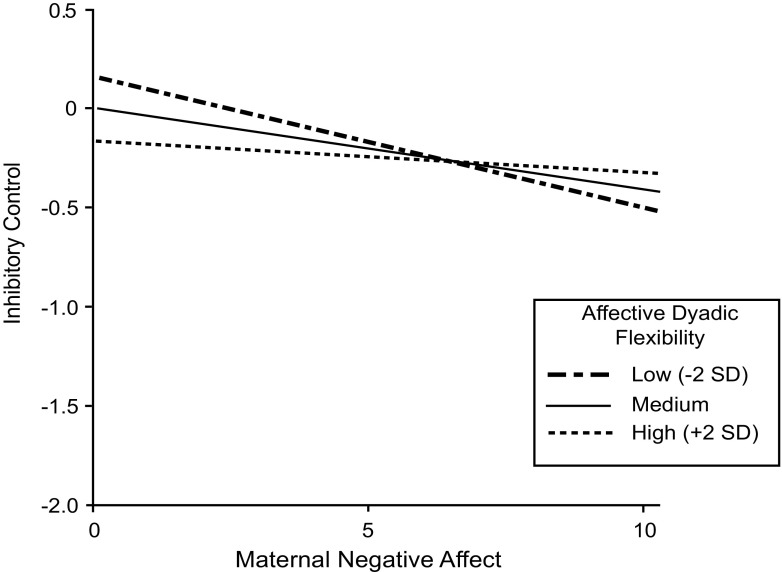
Plot of the interaction effect of ‘affective dyadic flexibility x negative affect mother’ on inhibitory control in preschoolers 9 months later. *Note.* Simple slopes for −1 SD and mean were significantly different from zero, as *b* = −0.08, *p* = 0.011, and *b* = −0.05, *p* = 0.016, respectively. The simple slope for +1 SD was not significantly different from zero: *b* = −0.02, *p* = 0.167

Third, when inspecting the estimated coefficient of the interaction term between affective dyadic flexibility and maternal negative affect (H3), results showed that the structure and the content of mother-child interactions indeed interact in predicting inhibitory control in preschoolers 9 months later. As shown in Fig. 3, higher levels of maternal negative affect were associated with lower levels of inhibitory control, but the relation was stronger for mother-child dyads who showed low levels of affective dyadic flexibility, hence more affectively rigid dyads. The indirect effect of this interaction was also significant (*B* = −0.11, *SE B* = 0.05, β = −0.17, *p* = 0.036).

## Discussion

In the current study, we examined whether preschoolers’ inhibitory control operates as a mechanism underlying the association between mother-child interactions and hyperactive/impulsive behavior and aggressive behavior over time. By using a DS approach we were able to explore the role of both the content (i.e., maternal negative affect) of mother-child interactions, as well as its dyadic structure (i.e., affective dyadic flexibility).

### Hyperactive/Impulsive Behavior

Our results indicated that the relation between maternal negative affect and children’s hyperactivity/impulsivity was indeed mediated by preschoolers’ inhibitory control even after taking into account their initial levels of hyperactivity/impulsivity. More specifically, mother-child interactions characterized by higher levels of maternal negative affect were associated with lower levels of inhibitory control in preschoolers 9 months later, which ultimately related to elevated levels of hyperactive/impulsive behavior another 9 months later. Our findings are in line with the cross-sectional study by Eisenberg et al. (2001), suggesting that children are less able to learn in a negative environment, and have trouble internalizing cognitive processes such as inhibitory control, resulting in more hyperactive/impulsive behavior problems. With our study we provided longitudinal support for this theory.

Similar results were found for the indirect effect of affective dyadic flexibility: Mother-child interactions with higher levels of affective dyadic flexibility were associated with lower levels of inhibitory control in preschoolers 9 months later, which ultimately related to more hyperactive/impulsive behavior as reported by teachers another 9 months later. This was after controlling for initial hyperactivity/impulsivity of preschoolers. These findings emphasize the role of mothers as an external regulator of affect during the preschool years (Bernier et al. 2010; Calkins et al. 1998), through which children can acquire the needed cognitive skills (i.e., inhibitory control) to gradually develop the ability to self-regulate. The relation between affective dyadic flexibility and children’s adjustment, however, depends on children’s age and their cognitive development, since older children seem to benefit from more flexible mother-child interactions (De Rubeis and Granic 2012; Granic et al. 2007; Hollenstein et al. 2004), whereas our findings suggest that preschoolers show less hyperactivity/impulsivity when mother-child interactions are rigid. Based on this finding, we believe that a change in conceptualization of affective dyadic flexibility in mother-child dyads during the preschool years is appropriate. Rather than referring to affectively flexible versus rigid mother-child interactions, we suggest to use the term affective dyadic instability versus affective dyadic stability. Future research should examine whether there is a specific age or developmental stage at which affectively stable mother-child interactions switch from predicting less to more adjustment problems in children and more importantly, why this might be the case.

Furthermore, affective dyadic instability was found to interact with maternal negative affect in predicting inhibitory control, and indirectly also predicted preschoolers’ hyperactive/impulsive behavior. The negative association between maternal negative affect and children’s inhibitory control is stronger for dyads that are highly stable. Thus, on the one hand the results support the idea that preschoolers would benefit from more affectively stable interactions with their mother. On the other hand, the detrimental effect of maternal negativity might become more pronounced when this occurs in highly stable, predictable interaction patterns between mothers and their preschool children. Although more research is needed, these findings emphasize the interplay between the content of a mother-child interaction and the structure in which it is imbedded.

### Aggressive Behavior

In contrast to hyperactive/impulsive behavior, our hypothesized predictors did not directly nor indirectly relate to preschoolers’ aggressive behavior after controlling for initial aggressive behavior. One explanation for this could be that aggressive behavior may be too stable over time to reveal statistical significant predictors, as initial aggressive behavior scores were strongly correlated with aggressive behavior 18 months later, whereas hyperactivity/impulsivity showed a moderate association between the first assessment and at the 18-month follow-up (see Table 1; Cohen 1988).

A second explanation for our inability to predict aggressive behavior could be that regulating and inhibiting this behavior requires a different type of inhibitory control than the one needed to inhibit hyperactive/impulsive behavior. Suppressing aggressive behavior could demand cognitive control in a more emotionally-laden situation, whereas the inhibition of hyperactive/impulsive behaviors would require emotionally neutral cognitive processes. Previous research supports the need to differentiate between hot and cool cognitive aspects of inhibitory control (Hongwanishkul et al. 2005). As we already noted, associations between inhibitory control and aggressive behavior are more profound when motivational demands, such as reward and punishment are high (Fairchild et al. 2009; Matthys et al. 2004; Schoemaker et al. 2012). In the current study, inhibitory control tasks predominantly required cool cognitive skills, which could explain the inability of our model to predict preschoolers’ aggressive behavior.

Third, the lack of significant findings regarding preschoolers’ aggressive behavior might be explained by the way aggressive behavior was measured in our study. Tremblay (2000) has already pointed out that a number of items on the CBCL/TRF Aggression scale (Achenbach and Rescorla 2000) do not specifically refer to aggressive behavior (e.g., wants attention, selfish). This may have affected the results.

## Conclusions

Taken together, our findings are in line with the recent work of Sulik et al. (2015) and demonstrate that inhibitory control acts as a mechanism linking mother-child interactions to preschoolers’ hyperactivity and impulsivity over time. That is, longitudinal associations between both the content and structure of mother-child interactions and later hyperactive/impulsive behavior problems were mediated by preschoolers’ inhibitory control. Our use of SSGs (e.g., micro approach) in unraveling mother-child interactions adds to the strength of the study, as it seems to be an improvement over and above using global measures (e.g., macro approach). By disentangling the affective content from the affective dyadic structure in mother-child interactions, this study adds to previous knowledge by demonstrating that both characteristics are important for child development. Moreover, the role of maternal negative affect seems to be dependent on the structure of the mother-child interaction it is imbedded in. This underscores the unique contribution of using micro ratings in unraveling dyadic parent-child dynamics in relation to child development.

Hence, based on independent measures, our findings provide support for a process model in which affectively stable mother-child interactions that are low in maternal negative affect promote young children’s inhibitory control, which in turn reduces children’s hyperactive/impulsive behavior problems. These results were found even when accounting for initial hyperactive/impulsive behavior problems and after controlling for children’s medication intake or psychosocial treatment. This conclusion is based on a sample of predominantly clinically referred preschoolers, thus children who experience severe levels of hyperactivity and impulsivity. Our results also emphasize the importance of differentiating between hyperactive/impulsive behavior and aggressive behavior when targeting externalizing problem behaviors in preschool children.

### Limitations

Our findings provide relevant information for children who show hyperactive/impulsive behavior problems at the clinical level. However, the conclusions should also be considered in the light of the following limitations. First, due to a small number of girls in our sample, we were unable to test whether the examined relations might vary across gender, which should be addressed by future research. Second, the operationalization of aggressive behavior in preschoolers was not optimal (e.g. Tremblay 2000). Third, future studies might consider specifically targeting inhibitory control tasks that require hot cognitive skills in order to examine preschoolers’ aggressive behavior. Fourth, in the current design we were unable to test for bidirectional effects of mother-child interaction patterns, inhibitory control, and children’s problem behavior. Moreover, it should be noted that we did not control for previous inhibitory control skills of preschoolers. Future research should test such a “full” longitudinal model (i.e., with all constructs – predictor, mediator and outcome – assessed at all measurement moments), with a more appropriate sample size for such a complex model.

### Clinical Implications

Our findings emphasize the relevance of mother-child interactions in predicting preschoolers’ hyperactivity/impulsivity. In this study we demonstrated how affectively stable mother-child interactions and low levels of maternal negative affect are important in the promotion of preschoolers’ inhibitory control, and indirectly in reducing hyperactive/impulsive behavior problems in children that display these problems at a clinical level.

Intervention programs aimed at reducing externalizing behavior problems in young children already target the affective responses of parents (i.e., PCIT, Zisser and Eyberg 2010; and Incredible Years, Webster-Stratton 2011). Our results further support the clinical relevance of this for hyperactive and impulsive behavior problems. This is especially noteworthy as the effect of parent training programs in the treatment of ADHD and ADHD symptoms has not been convincing in previous research (e.g., Daley et al. 2014).

The current study thus supports the need for further examination of parent training programs for the treatment of ADHD symptoms in young children diagnosed with ADHD and/or ODD/CD, under the condition that the intervention also focuses on achieving affectively stable mother-child interactions that are low in maternal negativity. Lastly, our findings also indicate interventions should give distinct attention to the development of inhibitory control in preschoolers as it operates as a mechanism that links the interactive behavior between mothers and their preschool children to positive child development.

## Electronic supplementary material

ESM 1 (DOCX 21.5 kb)

## Footnotes

1 In the present study we focus on negative affect, because the observational setting was specifically designed to provoke a potential stressful situation (i.e., the mother and child had to work on the tasks, while attractive toys were available, but the child was not allowed to play with them). Maternal positive affect was coded as well, but there were hardly any relations between positive affect and the other measures (i.e., affective dyadic flexibility T1, inhibitory control measures T2, C-TRF/1.5–5 measures T3). For these reasons we decided to exclude this measure from the present study.
