# Supplementary material for: Mother-Child Interactions and Externalizing Behavior Problems in Preschoolers over Time: Inhibitory Control as a Mediator
Source: J Abnorm Child Psychol. 2017 Jan 31;45(8):1503–17. doi: 10.1007/s10802-016-0258-1 (PMC5655588; doi:10.1007/s10802-016-0258-1)
Supplement: Supplementary file 1 — (DOCX 21.5 kb) [file 10802_2016_258_MOESM1_ESM.docx]

**Supplementary material**

The report of the Bayesian estimates of our model was proceeded by a thorough check of convergence, using the WAMBS-checklist (Depaoli & Van de Schoot 2015). Starting values based on the ML-estimates were used and two Markov chains were implemented for each parameter. Furthermore, we used a seed value of 8 for the analyses. The Brooks, Gelman, and Rubin (BGR) convergence diagnostic was applied as described in the *Mplus* manual, but a stricter convergence criterion of 0.01 rather than the default setting of 0.05 was used, as suggested by Depaoli and Van de Schoot (2015). We specified an initial burn-in phase of 500,000 iterations, with a fixed number of post burn-in iterations of also 500,000. The number of iterations were established after checking the BGR diagnostic and visually inspecting trace plots for each model parameter (i.e., both Markov chains had to be visually stacked with a constant mean and variance in the post burn-in portion of the chain).

To guarantee that convergence to the target distribution was obtained and local convergence was not an issue, the model was estimated again with a burn-in phase of 1,000,000 and 1,000,000 post burn-in. Again, the BGR indicated that convergence was obtained and inspection of the plots remained consistent with that result. The percent bias for model parameters obtained in the two analyses revealed that results were almost identical with bias levels less than |1|% for nearly each of the model parameters. Three parameter estimates did exceed the bias level of |1|%, but this was due to the small value of the estimates (e.g., -.079 and -.078), for which differences in estimates of only Δ*b* =  .001 are already postulated as biased at the 1% level. Hence, these differences in estimates were not considered as problematic and our post burn-in value of 1,000,000 was deemed sufficient.

**Results**

       The posterior predictive *p*-value (PPP) was .275, indicating that our specified model adequately fits the data (Gelman, Meng, & Stern, 1996). The median estimates and the 95% Bayesian credibility intervals are reported in Table 3. A credibility interval refers to probability of the population regression coefficient falling within this interval. When the credibility interval does not contain the value of 0, it is indicated that the regression coefficient of interest is likely to represent a non-zero effect (Depaoli & Van de Schoot, 2015). Results yielded similar patterns of effects as the frequentist approach, using the MLR estimation method.

Table 3

*Median Estimates and Associated Credibility Intervals (Bayesian Estimation; N = 173)*

|  | *Mdn* | 95% CI | |
| --- | --- | --- | --- |
|  |  | Lower CI | Upper CI |
| Paths a      Aff. dyadic flexibility T1   🡪 Inhibitory control T2      Negative affect mother T1  🡪 Inhibitory control T2      Flex*Neg T1                     🡪 Inhibitory control T2  Paths b      Inhibitory control T2        🡪 Hyperactive/impulsive T3      Inhibitory control T2        🡪 Aggressive behavior T3  Paths c’      Aff. dyadic flexibility T1 🡪 Hyperactive/impulsive T3      Negative affect mother T1  🡪 Hyperactive/impulsive T3      Flex*Neg T1                     🡪 Hyperactive/impulsive T3      Aff. dyadic flexibility T1     🡪 Aggressive behavior T3      Negative affect mother T1  🡪 Aggressive behavior T3      Flex*Neg T1                     🡪 Aggressive behavior T3  Stability measures      Hyperactive/impulsive T1 🡪 Hyperactive/impulsive T3      Aggressive behavior T1 🡪 Aggressive behavior T3  Covariances      Aff. dyadic flexibility T1    ↔ Hyperactive/impulsive T1      Negative affect mother T1  ↔ Hyperactive/impulsive T1  Aff. dyadic flexibility T1    ↔ Negative affect mother T1      Aff. dyadic flexibility T1    ↔ Aggressive behavior T1      Negative affect mother T1 ↔ Aggressive behavior T1      Hyperactive/impulsive T1 ↔ Aggressive behavior T1      Hyperactive/impulsive T3 ↔ Aggressive behavior T3 | **-.11**  **-.05**  **.01**    **-8.10**  -1.95    -.37  -.15  .12  .01  .21  .04    **.24**  **.35**    **6.26**  **18.11**  **7.09**  **5.84**  **20.82**  **89.88**  **28.07** | **-.20**  **-.09**  **.01**    **-32.15**  -10.31    -3.21  -1.39  -.03  -1.11  -.30  -.08    .**14**  **.25**    **2.42**  **7.26**  **5.12**  **2.14**  **10.46**  **67.32**  **15.66** | **-.05**  **-.02**  **.03**    **- 2.92**  2.67    .64  .32  .49  .84  .61  .20    **.35**  **.44**    **10.69**  **30.62**  **9.64**  **10.09**  **33.11**  **119.62**  **40.25** |

*Note.* Positive effects are in boldface. *Mdn* = Median; CI = Credibility Interval.

**References**

Depaoli, S., & Van de Schoot, A.G.J. (2015). Improving transparency and replication in Bayesian statistics: The WAMBS-Checklist. *Psychological Methods*. *Advance online publication.* doi:10.1037/met0000053

Gelman, A., Meng, X. L., & Stern, H. (1996). Posterior predictive assessment of model fitness via realized discrepancies. *Statistica Sinica, 6*, 733-760. Retrieved from http://www.jstor.org/stable/24306036
